# Secular trends and age-period-cohort effect on adverse perinatal outcomes in Hubei, China (2011–2019)

**DOI:** 10.1038/s41598-022-27194-8

**Published:** 2022-12-29

**Authors:** Hui Li, Yuanmei Shi, Zahoor Ahmed, Abbas khan, Kang Xu, Xiaoping Yin, Nawsherwan, Hong Zhang

**Affiliations:** 1 Department of Medicine, Taixing People Hospital, Taizhou, Jiangsu China; 2 Department of Pediatrics, Taixing People Hospital, Taizhou, Jiangsu China; 3 School of Food and Agricultural Sciences, University of Management and Technology, Lahore, Pakistan; 4 Department of Nutrition and Health Promotion, University of Home Economics, Lahore, Pakistan; 5 Department of Preventive Medicine, School of Health Sciences, Wuhan University, Wuhan, Hubei China; 6 Xiamen Cardiovascular Hospital of Xiamen University, School of Medicine, Xiamen University, Xiamen, China

**Keywords:** Health care, Medical research

## Abstract

The increasing trend in the incidence of adverse perinatal outcomes is a public health concern globally as well as in China. However, the causes of the increasing trend are not well understood. The present tertiary-hospital-based retrospective study (2011–2019) aims to determine the secular trends and age-period-cohort effect on adverse perinatal outcomes in Hubei, China. The age-standardized incidence rates of adverse perinatal outcomes significantly decreased such as preterm births by 22% [AAPC − 3.4% (95% CI − 7.8, − 1.2)], low birth weight (LBW) by 28.5% [AAPC − 4.7% (95% CI − 6.0, − 3.3)], and fetal distress by 64.2% [AAPC − 14.0% (95% CI − 17.8, − 10.0)] during 2011–2019. Both extremes of maternal age groups (18–20 years and 42–44 years) had a higher risk ratio for adverse perinatal outcomes including preterm birth, perinatal mortality, LBW, low ponderal index (LPI), low Apgar score, and congenital defect compared to the reference age group (30–32 years). A higher risk ratio for perinatal mortality, intrauterine growth restriction (IUGR), and fetal distress and a lower risk ratio for preterm births and LBW were observed in the period 2017–2019. Both the young cohort (1997–1999) and the old cohort (1976–1969) had a higher risk ratio for preterm birth, perinatal mortality, macrosomia, and congenital defect compared to the reference cohort (1982–1984). In conclusion, some of the adverse perinatal outcomes incidence significantly decreased in the last 9 years in Hubei. However, extremes of maternal age groups and both young and old cohorts were associated with a higher risk of preterm birth, perinatal mortality, and congenital defect.

## Introduction

Adverse perinatal outcomes are the unfavorable results of pregnancy that include perinatal mortality, preterm births, and babies with low birth weight (LBW)^1^. These adverse perinatal outcomes are the most common pregnancy outcomes in developing counties and have a significant impact on infants, families, and communities^2^.

Globally, perinatal mortality (0–28 days) contributing 43% in under-five years of deaths each year and preterm birth rates increased from 9.8% in 2000 to 10.6% in 2014. However, LBW declined from 17.5% in 2000 to 14.6% in 2015. The trends and incidence rates of adverse perinatal outcomes vary in different countries^3–5^. The increasing trend of preterm births rate^6–8^ and decreasing trend of perinatal mortality and LBW rate have been observed in different regions and provinces of China^9–12^.

Neonates with adverse perinatal outcomes are at higher risk for mortality and various short and long-term health consequences. Preterm babies are associated with an increased risk of early life death, and long-term health problems including mental retardation, cerebral palsy, visual and hearing impairments, and poor health and growth^13^. LBW is associated with a higher risk of non-communicable diseases later in life^13^. Adverse perinatal outcomes are mostly linked to maternal factors, the majority of which can be avoided with the right care during pregnancy, childbirth, and the postpartum period^14^. China’s government health policies and economic development have improved the quantity and quality of maternal, child, and newborn health care over the last two decades^15^.

Several epidemiological studies have observed the trend in adverse perinatal outcomes in different regions of China. However, heterogeneity exists in the findings of their studies. Moreover, no or limited studies highlighted the secular trend and age-period-cohort effect on adverse perinatal outcomes in Hubei, China. Hubei province is a large economically and ethnically diversified province located in south-central China with approximately 57 million people^8^. The aim of this study was therefore to determine the secular trend and age-period-cohort effect on adverse perinatal outcomes in Hubei, China from 2011 to 2019.

## Results

### General maternal-neonatal characteristics across different maternal age groups

Among the total pregnant women (N = 23,085), 49.2% (n = 11,354) were < 30 years of age and 50.8% (n = 11,731) were ≥ 30 years old. Compared to the reference age group (30–32 years), both extremes of maternal age groups (18–20 years) and (42–44 years) had a significantly higher incidence of hypertensive disorders of pregnancy (HDP), preterm birth, perinatal mortality, LBW, LPI, and low Apgar score (Table 1).

**Table 1:** Distribution of maternal-neonatal traits across different maternal age groups (N = 23,085).

Maternal-neonatal traits	Groups of maternal age									
	18-20yrs (n = 198) No. (%)	21-23yrs (n = 1009) No. (%)	24-26yrs (n = 3467) No. (%)	27-29yrs (n = 6680) No. (%)	30-32yrs (n = 5436) No. (%)	33-35yrs (n = 3226) No. (%)	36-38yrs (n = 1877) No. (%)	39-41yrs (n = 870) No. (%)	42-44yrs (n = 322) No. (%)	*p*-value
**Parity**										
Primiparous (≤ 1)	180 (90.9)	882 (87.4)	2964 (85.5)	5620 (84.1)	4072 (74.9)	2101 (65.1)	1090 (58.1)	448 (51.5)	157 (48.8)	< 0.001
Multiparous (> 1)	18 (9.1)	127 (12.6)	503 (14.5)	1060 (15.9)	1364 (25.1)	1125 (34.9)	787 (41.9)	422 (48.5)	165 (51.2)	
C-section*	85 (42.9)	463 (45.9)	1735 (50.0)	3747 (56.1)	3418 (62.9)	2292 (71.0)	1372 (73.1)	661 (76.0)	246 (76.4)	< 0.001
Previous history of C-section*	14 (7.1)	49 (4.9)	267 (7.7)	662 (9.9)	914 (16.8)	796 (24.7)	563 (30.0)	252 (29.0)	77 (23.9)	< 0.001
HDP*	13(6.5)	61(6.1)	210(6.1)	351(5.2)	346(6.4)	267(8.2)	131(6.9)	101(11.6)	47(14.6)	< 0.001
Abnormal* placentation	5(2.5)	35(3.4)	120(3.4)	249(3.7)	229(4.2)	182(5.6)	102(5.4)	71(8.1)	25(7.7)	< 0.001
GDM*	5(2.5)	17(1.7)	144(4.2)	354(5.3)	389(7.2)	286(8.9)	220(11.7)	83(9.5)	40(12.4)	< 0.001
**Perinatal outcomes**										
Preterm birth*	60(30.3)	274(27.2)	678(19.6)	1021(15.3)	907(16.7)	679(21.0)	439(23.4)	257(29.5)	115(35.7)	< 0.001
Perinatal* mortality	12(6.1)	15(1.5)	55(1.6)	75(1.1)	58(1.1)	42(1.3)	40(2.1)	24(2.8)	12(3.7)	< 0.001
LBW*	53(26.8)	236(23.4)	560(16.2)	749(11.2)	668(12.3)	453(14.0)	311(16.6)	178(20.5)	75(23.3)	< 0.001
IUGR*	1(0.5)	8(0.8)	26(0.7)	54(0.8)	39(0.7)	19(0.6)	10(0.5)	7(0.8)	4(1.2)	0.8
LPI*	14(7.1)	67(6.6)	143 (4.1)	214(3.2)	210(3.9)	119(3.7)	79(4.2)	33(3.8)	19(5.9)	< 0.001
Low Apgar score*	23(11.6)	53(5.3)	133(3.8)	206(3.1)	155(2.9)	123(3.8)	80(4.3)	45(5.2)	26(8.1)	< 0.001
Fetal distress*	7(3.5)	23(2.3)	75(2.2)	151(2.3)	132(2.4)	67(2.1)	41(2.2)	17(2.0)	9(2.8)	0.8
Fetal* macrosomia	2(1.0)	27(2.7)	153(4.4)	377(5.6)	338(6.2)	183(5.7)	109(5.8)	45(5.2)	18(5.6)	< 0.001
^a^Congenital defects*	6(3.0)	17(1.7)	47(1.4)	89(1.3)	52(1.0)	49(1.5)	19(1.0)	12(1.4)	7(2.2)	0.05
**Neonatal gender**										
Male	107 (54.0)	545 (54.0)	1867 (53.9)	3489 (52.2)	2892 (53.2)	1792 (55.5)	1004 (53.5)	456 (52.4)	188 (58.4)	0.08
Female	91 (46.0)	464 (46.0)	1600 (46.1)	3191 (47.8)	2544 (46.8)	1434 (44.5)	873 (46.5)	414 (47.6)	134 (41.6)	

*HDP* Hypertensive disorders of pregnancy, *GDM* Gestational diabetes mellitus, *LBW* Low birth weight, *IUGR* Intrauterine growth restriction, *LPI* Low ponderal index, low Apgar score (< 7).

*Frequency and percentage of variables with only ‘Yes’ value presented.

^a^Congenital defects (microtia, anotia, polydactyly, heart defects, limb reduction defects, cleft lip, cleft palate, hydrocephaly, and NTDs), *p-*values were calculated using chi-square test.

### Secular trends of adverse perinatal outcomes from 2011 to 2019

**Figure 1:**
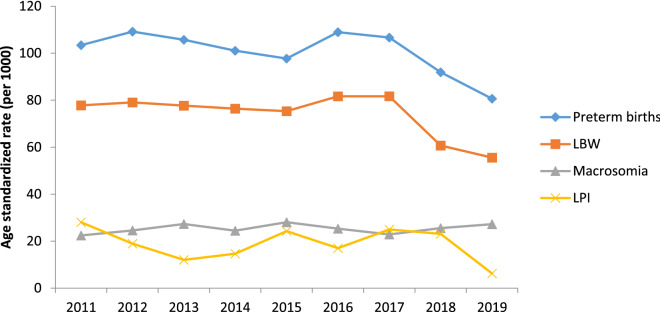
Trend of adverse of perinatal outcomes across different years of study (2011–2019).

**Figure 2:**
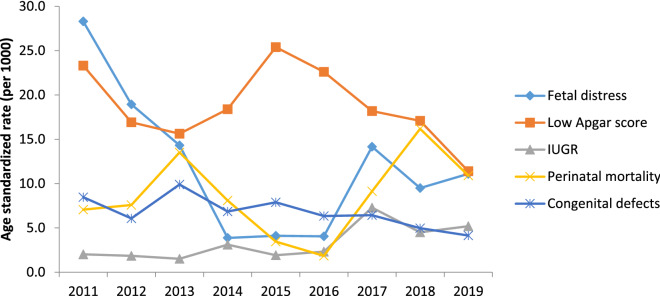
Trend of adverse perinatal outcomes across different years of study (2011–2019).

Based on joinpoint regression analysis, age-standardized incidence rates of adverse perinatal outcomes significantly decreased such as preterm births by 22% [AAPC − 3.4% (95% CI − 7.8, − 1.2)], LBW by 28.5% [ AAPC − 4.7% (95% CI − 6.0, − 3.3)], and fetal distress by 64.2% [AAPC − 14.0% (95% CI − 17.8, − 10.0)] during 2011–2019. On the other hand, age-standardized incidence rates of perinatal mortality non-significantly increased by 53.5% [AAPC 13.7% (95% CI − 32.7, 92.2)], IUGR by 160% [AAPC 12.0% (95% CI − 19.7, 56.4)], and macrosomia by 21.4% [AAPC 2.6% (95% CI − 10.0, 16.9) during the study period [Tables 2, 3, Figs. 1 and 2].

**Table 2:** Trends of adverse perinatal outcomes (preterm births, perinatal mortality, LBW, IUGR, and LPI) using joinpoint regression analysis from 2011–2019.

Variables and segments	Year	APC (95% CI)
**Preterm births**		
Trend1	2011–2014	− 1.6 (− 27.8, 34.2)
Trend2	2014–2017	1.8 (− 45.3, 89.2)
Trend3	2017–2019	− 13.2 (− 53.3, 61.3)
AAPC (95% CI)	2011–2019	− 3.4 (− 7.8, − 1.2)*
**Perinatal mortality**		
Trend1	2011–2013	35.9 (− 90.9,179.2 )
Trend2	2013–2016	− 39.5 (− 99.9, 662.9)
Trend3	2016–2019	89.8 (− 94.2, 689.3)
AAPC (95% CI)	2011–2019	13.7 (− 32.7, 92.2)
**LBW**		
Trend1	2011–2014	− 1.2 (− 10.0, 8.5)
Trend2	2014–2017	1.7 (− 15.6, 22.6)
Trend3	2017–2019	− 18.0 (− 32.0, − 1.1)*
AAPC (95% CI)	2011–2019	− 4.7 (− 6.0, − 3.3)*
**IUGR**		
Trend1	2011–2013	− 12.3 (− 99.4, 123.9)
Trend2	2013–2017	32.0 (− 89.1, 151.9)
Trend3	2017–2019	3.2 (− 99.3,101.3 )
AAPC (95% CI)	2011–2019	12.0 (− 19.7, 56.4)
**LPI**		
Trend1	2011–2013	− 33.4 (− 99.8, 54.8)
Trend2	2013–2017	22.6 (− 93.8, 234.8)
Trend3	2017–2019	− 48.3 (− 99.9, 30.9)
AAPC (95% CI)	2011–2019	− 15.2 (− 43.1, 26.4)

*APC* annual percentage change, *APPC* average annual percent change, *CI* confidence interval, *LBW* Low birth weight, *IUGR* Intrauterine growth restriction, *LPI* Low ponderal index.

*Significantly different from 0 at alpha = 0.05 (*p* < 0.05).

**Table 3:** Trends of adverse perinatal outcomes (Low Apgar score, fetal distress, macrosomia, and congenital defects) using joinpoint regression analysis from 2011–2019.

Variables and segments	Year	APC (95% CI)
**Low Apgar score**		
Trend1	2011–2013	− 15.1 (− 94.7, 126.0)
Trend2	2013–2016	16.5 (− 92.7, 167.3)
Trend3	2016–2019	− 22.5 (− 80.6, 210.2)
AAPC (95% CI)	2011–2019	− 7.6 (− 25.0, 13.8)
**Fetal distress**		
Trend1	2011–2014	− 51.4 (− 64.1, − 34.2)*
Trend2	2014–2017	28.8 (− 29.6, 135.8)
Trend3	2017–2019	10.5 (− 39.6, 102.2)
AAPC (95% CI)	2011–2019	− 14.0 (−17.8, −10.0)*
**Macrosomia**		
Trend1	2011–2013	10.0 (− 84.5, 82.8)
Trend2	2013–2017	− 2.8 (− 63.6, 19.2)
Trend3	2017–2019	6.5 (− 85.0, 57.5)
AAPC (95% CI)	2011–2019	2.6 (− 10.0, 16.9)
**Congenital defects**		
Trend1	2011–2013	4.9 (− 93.4, 57.7)
Trend2	2013–2017	− 6.8 (− 76.6, 70.5)
Trend3	2017–2019	− 19.0 (− 94.9, 80.4)
AAPC (95% CI)	2011–2019	− 7.3 (− 22.9, 11.5)

*APC* annual percentage change, *APPC* average annual percent change, *CI* confidence interval.

*Significantly different from 0 at alpha = 0.05 (*p* < 0.05).

### Secular trends of HDP and abnormal placentation from 2011 to 2019

Age-standardized rates of HDP significantly increased by 2.7% [AAPC 2.5% (95% CI 1.1, 3.9)] and abnormal placentation by 21% [AAPC 1.2% (95% CI 1.1, 2.1)] during the study period (Table S1 and Fig. S1).

### Age-period-cohort effect

#### Age effect

After controlling for period and cohort effects, both extremes of maternal age groups had a higher risk ratio for adverse perinatal outcomes including preterm birth, perinatal mortality, LBW, LPI, low Apgar score, and congenital defect compared to the reference age group (Tables 4 and 5).

**Table 4:** Age-Period-Cohort effects on preterm births, perinatal mortality, LBW, IUGR and LPI.

Variables	Adverse perinatal outcomes (RR 95% CI)				
	Preterm births	Perinatal mortality	LBW	IUGR	LPI
**Age**					
18–20	1.82 (1.77, 1.84)	2.43 (2.29, 3.12)	1.98 (1.92, 2.02)	0.52 (0.37, 0.76)	1.51 (1.46, 1.56)
21–23	1.53 (1.48, 1.57)	1.18 (1.11, 1.52)	1.68 (1.61, 1.74)	1.90 (1.74, 2.18)	1.49 (1.38, 1.59)
24–26	1.06 (1.04, 1.08)	0.97 (0.92, 1.23)	1.14 (1.10, 1.17)	1.81 (1.64, 2.12)	0.95 (0.91, 0.98)
27–29	0.85 (0.84, 0.85)	0.72 (0.73, 0.85)	0.84 (0.83, 0.84)	1.59 (1.50, 1.79)	0.77 (0.76, 0.77)
30–32	1.00	1.00	1.00	1.00	1.00
33–35	1.38 (1.34, 1.39)	0.86 (0.89, 1.03)	1.24 (1.22, 1.24)	0.93 (0.92, 0.99)	1.00 (1.00, 1.01)
36–38	1.49 (1.45, 1.54)	2.99 (2.75, 3.80)	1.39 (1.34, 1.42)	1.16 (1.18, 1.20)	1.09 (1.04, 1.14)
39–41	1.77 (1.70, 182)	1.49 (1.39, 1.95)	1.56 (1.48, 1.61)	1.30 (1.18, 1.51)	0.89 (0.85, 0.92)
42–44	2.09 (2.04, 2.12)	3.51 (3.25, 4.60)	1.73 (1.67, 1.76)	1.80 (1.62, 2.08)	1.46 (1.42, 1.49)
**Period**					
2011–2013	1.05 (1.04, 1.05)	3.24 (2.79, 3.31)	1.03 (1.03, 1.04)	0.63 (0.60, 0.66)	1.03 (1.03, 1.04)
2014–2016	1.00	1.00	1.00	1.00	1.00
2017–2019	0.95 (0.94, 0.96)	4.42 (3.80, 4.51)	0.94 (0.93, 0.94)	3.01 (2.95, 3.09)	1.00 (0.99, 1.01)
**Cohort**					
1967–1969	1.14 (1.15, 1.16)	1.13 (1.06, 1.10)	0.90 (0.88, 0.92)	0.54 (0.37, 0.79)	0.93 (0.88, 0.96)
1970–1972	1.17 (1.13, 1.21)	1.36 (1.18, 1.38)	1.23 (1.20, 1.27)	2.51 (2.12, 2.99)	0.98 (0.95, 1.00)
1973–1975	1.10 (1.05, 1.15)	0.48 (0.41, 0.51)	1.26 (1.22, 1.33)	0.65 (0.54, 0.76)	1.41 (1.29, 1.53)
1976–1978	1.01 (0.98, 1.04)	0.98 (0.86, 1.00)	1.01 (0.99, 1.04)	0.75 (0.67, 0.82)	1.23 (1.17, 1.28)
1979–1981	1.01 (0.99, 1.03)	0.37 (0.34, 0.38)	1.00 (1.00, 1.01)	0.56 (0.52, 0.58)	1.08 (1.05, 1.10)
1982–1984	1.00	1.00	1.00	1.00	1.00
1985–1987	1.19 (1.17, 1.20)	0.74 (0.67, 0.76)	1.17 (1.16, 1.18)	1.06 (0.98, 1.14)	1.27 (1.24, 1.30)
1988–1990	1.23 (1.20, 1.27)	1.08 (0.98, 1.05)	1.25 (1.22, 1.29)	0.67 (0.57, 0.79)	1.35 (1.26, 1.44)
1991–1993	1.13 (1.08, 1.18)	1.37 (1.15, 1.45)	1.27 (1.21, 1.33)	0.69 (0.55, 0.83)	1.24 (1.12, 1.36)
1994–1996	0.91 (0.89, 0.94)	0.47 (0.44, 0.48)	1.02 (0.99, 1.05)	1.01 (0.85, 1.19)	1.24 (1.14, 1.32)
1997–1999	1.24 (1.22, 126)	2.42 (2.30, 2.52)	1.30 (1.27, 1.33)	2.89 (2.00, 4.14)	1.73 (1.67, 1.78)
**AIC**	9.78	7.51	9.53	5.49	7.47
**BIC**	4.11	16.49	5.37	− 7.98	− 13.60

*LBW* Low birth weight, *IUGR* Intra-uterine growth restriction, *LPI* Low ponderal index, *RR* risk ratio, *CI* confidence interval, *AIC* Akaike information criterion, *BIC* Bayesian information criterion.

**Table 5:** Age-Period-Cohort effects on low Apgar score, fetal distress, macrosomia, and congenital defect.

Variables	Adverse perinatal outcomes (RR 95% CI)			
	Low Apgar score	Fetal distress	Macrosomia	Congenital defect
**Age**				
18–20	4.52 (4.24, 4.83)	1.40 (1.36, 1.45)	0.13 (0.10, 0.16)	2.95 (2.67, 3.23)
21–23	1.57 (1.46, 1.70)	0.59 (0.58, 0.61)	0.50 (0.49, 0.52)	1.33 (1.18, 1.49)
24–26	1.06 (1.02, 1.12)	0.92 (0.90, 0.95)	0.97 (0.95, 0.99)	1.01 (0.92, 1.10)
27–29	0.90 (0.90, 0.93)	0.98 (0.97, 0.98)	0.99 (0.97, 0.99)	1.10 (1.04, 1.15)
30–32	1.00	1.00	1.00	1.00
33–35	1.46 (1.43, 1.51)	1.15 (1.15, 1.16)	0.82 (0.80, 0.83)	1.94 (1.85, 2.04)
36–38	1.83 (1.74, 1.96)	0.75 (0.73, 0.78)	0.78 (0.75, 0.80)	1.56 (1.46, 1.66)
39–41	1.56 (1.46, 1.70)	0.99 (0.98, 1.00)	0.69 (0.66, 0.71)	2.08 (1.86, 2.30)
42–44	2.58 (2.46, 2.78)	0.78 (0.77, 0.79)	0.64 (0.63, 0.64)	2.69 (2.54, 2.85)
**Period**				
2011–2013	0.81 (0.80, 0.82)	6.11 (5.89, 6.37)	0.88 (0.87, 0.88)	1.17 (1.17, 1.18)
2014–2016	1.00	1.00	1.00	1.00
2017–2019	0.71 (0.70, 0.72)	3.24 (3.18, 3.35)	0.99 (0.98, 0.99)	0.73 (0.71, 0.74)
**Cohort**				
1967–1969	0.33 (0.30, 0.36)	2.22 (2.13, 2.30)	1.78 (1.68, 1.86)	1.41 (1.41, 1.42)
1970–1972	1.83 (1.70, 1.97)	1.29 (1.28, 1.29)	1.52 (1.47, 1.56)	0.80 (0.73, 0.87)
1973–1975	0.88 (0.80, 0.97)	1.69 (1.57, 1.80)	1.05 (0.99, 1.10)	0.70 (0.63, 0.79)
1976–1978	0.94 (0.89, 1.01)	0.70 (0.70, 0.71)	1.01 (0.97, 1.05)	0.66 (0.62, 0.69)
1979–1981	0.82 (0.81, 0.84)	1.33 (1.29, 1.35)	1.11 (1.09, 1.12)	0.80 (0.79, 0.80)
1982–1984	1.00	1.00	1.00	1.00
1985–1987	1.12 (1.09, 1.14)	1.00 (0.98, 1.01)	0.76 (0.75, 0.77)	1.25 (1.22, 1.26)
1988–1990	1.40 (1.30, 1.50)	1.32 (1.25, 1.37)	0.84 (0.83, 0.84)	1.40 (1.29, 1.51)
1991–1993	1.18 (1.05, 1.33)	1.18 (1.09, 1.26)	0.43 (0.41, 0.46)	1.32 (1.13, 1.53)
1994–1996	0.57 (0.54, 0.61)	2.44 (2.33, 2.54)	1.25 (1.18, 1.31)	0.43 (0.42, 0.43)
1997–1999	1.12 (1.11, 1.13)	0.59 (0.49, 0.70)	2.01 (1.37, 2.92)	1.57 (1.51, 1.64)
**AIC**	8.68	6.89	7.33	6.31
**BIC**	18.84	− 6.66	− 14.49	− 14.84

*RR* risk ratio, *CI* confidence interval, *AIC* Akaike information criterion, *BIC* Bayesian information criterion.

#### Period effect

Compared to the reference period (2014–2016), a higher risk ratio for perinatal mortality, IUGR, and fetal distress and a lower risk ratio for preterm births and LBW was observed from 2017 to 2019 (Tables 4 and 5).

#### Cohort effect

Compared to the reference cohort (1982–1984), both the young cohort (1997–1999) and the old cohort (1967–1969) had a higher risk ratio for preterm birth, perinatal mortality, macrosomia, and congenital defect (Tables 4 and 5).

## Discussion

In the current tertiary hospital-based retrospective study (2011–2019), we showed secular trends in adverse perinatal outcomes and the age-period-cohort effect on adverse perinatal outcomes. Joinpoint regression analysis revealed that regardless of increasing secular trends of HDP and abnormal placentation (Table S1 and Fig. S1), age-standardized incidence rates of adverse perinatal outcomes significantly decreased such as preterm births, LBW, and fetal distress during the study period. Moreover, extremes of maternal age groups and both old and young cohorts were associated with a higher risk of adverse perinatal outcomes. A higher risk ratio for adverse perinatal outcomes was observed in the period 2017–2019.

Our findings indicated a significant declining trend in the age-standardized incidence rates of preterm births, LBW, and fetal distress from 2011 to 2019. In our study, the findings of decreasing trends in adverse perinatal outcomes are consistent with many previous studies^11,16–18^. To speculate, it might relate to several reasons, for example, rapid development in socioeconomic status (SES), improvement in maternal nutrition and health knowledge, higher access to the health care system, and improvement in neonatal rescue technology^19^.

An inverse relationship exists between maternal SES and adverse perinatal outcomes^20^. Women of higher SES status were associated with a lower incidence of LBW in Shaanxi, China^21^. Maternal SES strongly influences maternal nutrition^22^. During pregnancy, a nutritious diet (which contains adequate nutrients) and proper energy intake in each trimester allow proper fetal growth and decrease the risk of adverse perinatal outcomes^23^. An improved pregnancy outcome was found in women of optimum nutritional status in Chengdu, China^24^.

Health sector initiatives and investment had a great impact on maternal-neonatal health outcomes in China. It enables higher access to health care services and improvement in neonatal rescue technology. The Chinese government has increased health expenditure per capita from US$ 53 in 1995 to US$ 480 in 2012 and achieved remarkable goals in the last two decades. They have improved health workforce recruitment and training, health information systems and surveillance, and health insurance, resulting in a comprehensive three-tier medical and health service network extending from province to township and village level^25^.

Moreover, to better deliver and manage basic public health services, the Chinese government issued three editions of the National Basic Public Service Specifications in 2009, 2011, and 2017, respectively. These service packages of the program consist of health education, health management of children aged 0–6, and maternal health care^25^. Therefore, the strengthening and improvement of the three-tier medical and health services network for pregnant women in China has proven to decline the trends of adverse perinatal outcomes^26^.

Both extremes of maternal age groups had a higher risk ratio for preterm birth, perinatal mortality, LBW, LPI, low Apgar score, and congenital defects which is consistent with the previously reported studies^27,28^. Advanced maternal age (AMA) or maternal age over 40 years is associated with a higher risk of several adverse perinatal outcomes which is reported in various population-based studies^29–31^. In our study, women with AMA had a higher incidence of HDP, abnormal placentation, and GDM. These pregnancy complications in women with AMA could attribute to an increase in the risk of adverse perinatal outcomes in our study. In several previous cohort studies, women with HDP, abnormal placentation, and GDM had a higher risk to deliver LBW babies, preterm births, stillbirths, and perinatal mortality^32–35^.

Moreover, the higher risk of adverse perinatal outcomes in young women could be due to impaired vascular adaptation. Physical immaturity in adolescent mothers could hinder the physiological placental invasion through multiple pathways including an incomplete estrogen-dependent growth of the uterus, a residual ontogenetic progesterone resistance, and deficient tissue-specific programming of immune cells^28,36^.

We observed a higher risk ratio for perinatal mortality, IUGR, and fetal distress in the period 2017–2019. The increased risk of adverse perinatal outcomes in the period 2017–2019 would be explained by the announcement of China’s universal two-child policy (2015), the increased trend of women with AMA, the higher incidence rate of C-section, HDP, abnormal placentation, and multiparity^37^. The new China’s universal two-child policy may have increased the tendencies toward fertility desires among older women. These findings were also evidenced in the Chinese national surveillance data indicating that after the relaxation of the one-child policy, women with AMA increased from 7.8 to 10.9%^38^. In previous studies^39,40^, a substantial increase was observed in women with AMA after implementation of the China’s universal two-child policy. We also found that women with AMA increased from 12.5% in 2011–2013 to 21.1% in 2017–2019. Women with AMA could be a risk factor for increased risk of adverse perinatal outcomes in the period 2017–2019^37^.

We demonstrated that both old and young cohorts were associated with a higher risk of adverse perinatal outcomes. Over 7 decades ago, Baird^41^ proposed that many factors affect women’s early life-both in utero and postnatal which may affect their reproductive performance later in life. The higher risk of adverse perinatal outcomes in the old cohort (1967–1972) could be explained by poor health care facilities/services, lower socioeconomic status, and poor nutritional status of pregnant women in this cohort.

In China, the three-tier health care system was established in the 1950s and reversed the reforms in the 1980s. The new health care system experienced several constraints in this period. When the government centralized village-level health care facilities to the township level and various union clinics and cooperative health stations were centralized to communes; these changes resulted in deteriorating the quality and efficiency of health care services and reduced the availability and accessibility of health care services at village level^42,43^. Maternal lower socioeconomic status and poor nutritional status are associated with adverse perinatal outcomes^44–46^. The higher risk of adverse perinatal outcomes in the young cohort may be due to a remarkable change in lifestyle, higher stress due to rapid changes in the economic status of society, and increased environmental pollution^47–49^.

Our study has several limitations. The study period is comparatively short for finding secular trends and age-period-cohort analysis for adverse perinatal outcomes was not adjusted for parity, pre-pregnancy body weight, maternal education and occupation, and pregnancy complications. Our study was monocentric, which is a selection bias in this study. Moreover, due to a lack of data, we were unable to find secular trends in cause-specific adverse perinatal outcomes. As a tertiary-level hospital, many pregnant women with severe pregnancy complications are transferred to our hospital, resulting in a relatively high incidence of adverse perinatal outcomes. Therefore, our results cannot be generalized to the whole population and pregnant women living in other regions of China.

## Conclusion

In conclusion, regardless of increasing secular trends of HDP and abnormal placentation, the age-standardized incidence rates of adverse perinatal outcomes significantly decreased such as preterm births, LBW, and fetal distress during the study period. Moreover, extremes of maternal age groups and both old and young cohorts were associated with a higher risk of adverse perinatal outcomes. Higher risk ratios of adverse perinatal outcomes were observed in the period 2017–2019. This study would be useful to design planning and strategies to prevent increasing secular trends in HDP and abnormal placentation and implement adequate health care systems and intervention programs for reducing the burden of adverse perinatal outcomes in young and old pregnant women in Hubei, China.

## Material and methods

### Study population

A tertiary hospital-based retrospective study was conducted in the Wuhan University Renmin Hospital, Department of Obstetrics and Gynecology, Hubei, China from January 2011 to December 2019. The data was collected and documented in the obstetrics register and electronic database by trained nurses during individual examinations in the Gynecology and Obstetrics Department. The study protocol was approved by the Ethical Review Board of Renmin Hospital (ID: WDRY2019–K034) in accordance with the Declaration of Helsinki. The need for informed consent, according to national legislation, was waived by the Ethical Review Board of Renmin Hospital because this was a retrospective cohort study.

### Inclusion and exclusion criteria

**Figure 3:**
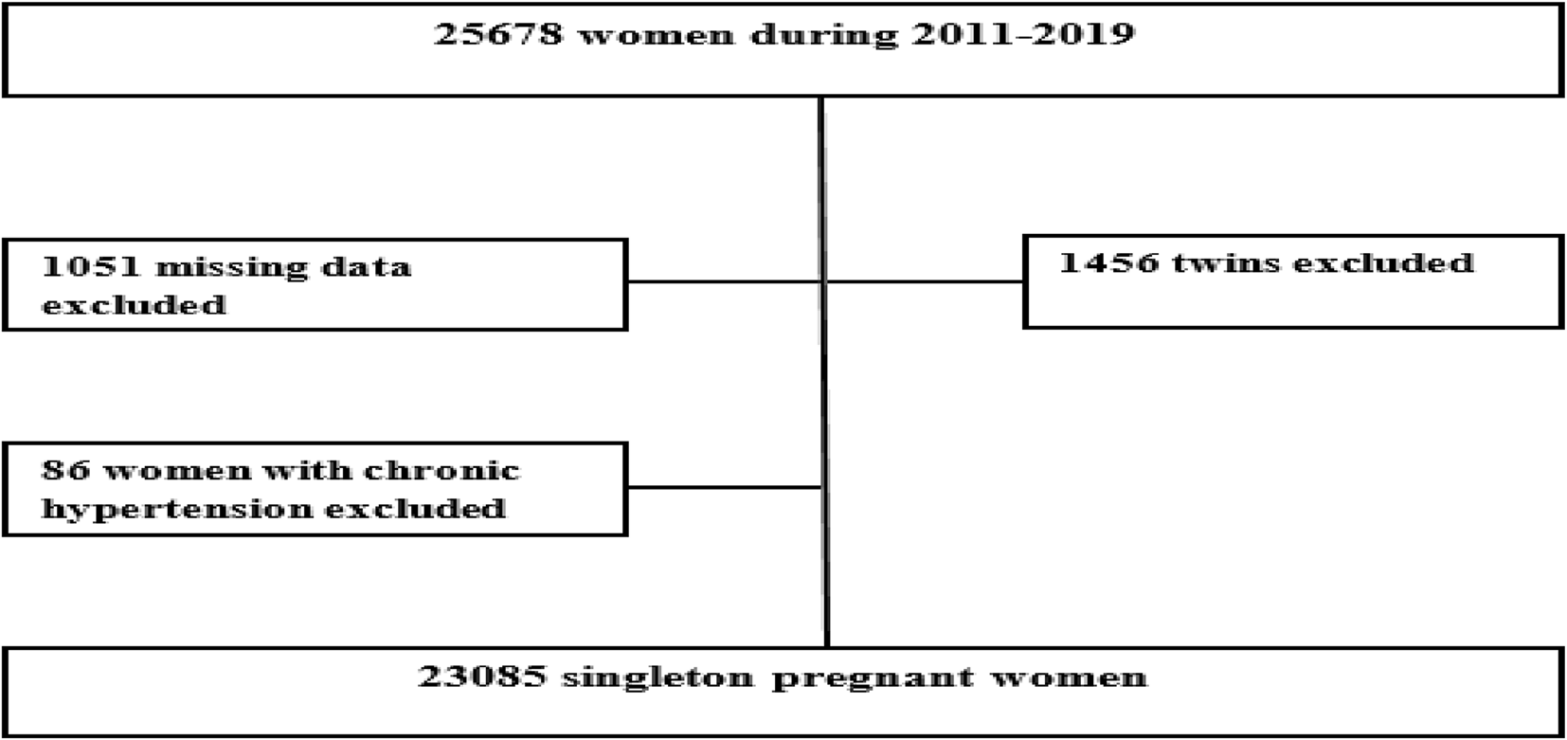
Flow chart of study population.

A total of 23,085 singleton pregnant women were selected for the study. We excluded missing data on maternal age, pre-pregnancy body weight, neonatal gender, birth weight, birth length, and gestational age^50^. Pregnant women with chronic hypertension and twin neonates were also excluded from the data analysis as shown in Fig. 3.

### Collection of data on maternal traits

Data regarding maternal traits were collected from the obstetrics register including maternal age, parity, pre-pregnancy body weight, gestational age, education, occupation, and pregnancy complications. Gestational age was calculated by the date of the last known menstrual period and confirmed by ultrasound examination during the first and second trimesters.

### Definition of perinatal birth outcomes

Neonatal birth outcomes were recorded immediately after neonatal birth including birth weight in grams using an electronic infant scale, and birth length in centimeters using a standard measuring board for the neonate. Preterm birth is defined as a neonate born before 37 completed weeks or fewer than 259 days from the first date of a woman’s last menstrual period^51^. Perinatal mortality is defined as the combination of late fetal mortality (stillbirths) and early neonatal mortality (0–6 days of life)^52^. Fetal macrosomia is defined as birth weight ≥ 4000 g and low birth weight (LBW) is defined as birth weight < 2500 g^53^. Intrauterine growth restriction (IUGR) is defined as a condition of fetal growth that is below the 10th percentile for its gestational age and does not reach its genetically predetermined growth potential^54^. Apgar score was determined by evaluating the newborn baby on five simple criteria on a scale from zero to two, then summing up the five values obtained. Apgar score was recorded at 1 min and 5 min after birth. Apgar score was divided into two categories (i) low Apgar score (< 7), and (ii) normal Apgar score (≥ 7)^55^. Fetal distress is defined as a pathophysiological condition in which the fetus is suffering from insufficient oxygen supply^56^. The ponderal index was determined by weight in gm / (length in cm)^3^ × 100. The ponderal index between 2.5 and 3.0 was considered normal between 2.0 and 2.5 marginal, and a neonate with a ponderal index less than 2.0 was considered a low ponderal index (LPI)^57^. Congenital defects are defined as abnormalities in the structure of neonatal body parts that occur during intrauterine development^58^.

### Statistical analysis

The categorical and binary variables are presented as number (n) and percentage (%). The Chi-square test was used to estimate the changes in general maternal-neonatal characteristics across various maternal age groups. *p* < 0.05 was taken as statistically significant. The data were analyzed using SPSS (Statistical Package for Social Sciences) for Windows version 22 (IBM Corporation, Chicago, USA).

The secular trend in adverse perinatal outcomes was estimated by joinpoint regression analysis. In the regression analysis, for each segment/period, the annual percentage changes (APC) and the average annual percentage changes (AAPC) in the rate of adverse perinatal outcomes were determined. The AAPC represents the trend in adverse perinatal outcomes in the whole period 2011–2019; while, APC indicates the trend in adverse perinatal outcomes in each segment/period identified by the joinpoint regression software. We presented the numbers of adverse perinatal outcomes change-points and estimated the model parameters by their associated *p-*values (< 0.05). Moreover, Monte Carlo methods were used to find each *p-*value and maintain the overall asymptotic significance level through Bonferroni correction^59^. This analysis was conducted using the joinpoint regression program version 4.8.0.1 (April 2020) from the Surveillance Research Program of the U.S. National Cancer Institute.

The aim of the age-period-cohort (APC) analysis is to estimate the effects of age, period, and cohort on adverse perinatal outcomes incidence. The age effect represents the association of adverse perinatal outcome incidence with different age groups. Period effect represents influencing factors, such as a series of historical events and environmental factors, and it reflects variation in the adverse perinatal outcomes incidence over time that influences all age groups simultaneously. The cohort effect shows variations of adverse perinatal outcomes incidence across groups of individuals born in the same year and changes in different lifestyles^60^. The common problem associated with the APC analysis is collinearity (i.e. birth cohort = period − age). The APC model is affected by the linearity between two variables, so it is impossible to determine the three independent linear APC variables of age, period, and cohort. We used the APC model with the intrinsic estimator (IE), which is a new method to estimate the coefficients and solve the collinearity problem by generating a distinctive set of trend estimates independent of any arbitrary assignment of identifying limitations on age, period, or cohort coefficients that may not be verified in the data itself^61^. Estimated coefficients for the age, period and cohort effects were produced by the APC analysis using the IE method. The exponential value [exp(coef.) = ecoef.] was created from these coefficients, which denotes the relative risk (RR) of a particular age, period, or birth cohort relative to the reference group.

In the APC model using the IE method, the age-specific adverse perinatal outcomes incidence rates were appropriately categorized into 9 age groups (18–20 years, 21–23 years, 24–26 years, 27–29 years, 30–32 years, 33–35 years, 36–38 years, 39–41 years, and 42–44 years). It has 3-years interval of periods (2011–213, 2014–2016, and 2017–2019) and 11 cohorts of birth (i.e. 1967–1969, 1970–1972, 1973–1975, 1976–1978, 1979–1981, 1982–1984, 1985–1987, 1988–1990, 1991–1993, 1994–1996, and 1997–1999). The general form of the APC model is written as Y = log (M) = μ + αage_1_ + βperiod_1_ + γcohort_1_ + ε; where, M is defined as the incidence rate in the age groups, α, β, and γ indicates the functions of age, period, and cohort effect, μ, and ε are the intercept item and the random error. The APC model was used to decompose the three trends and estimate efficient results^62^. Moreover, the Akaike information criterion (AIC), and Bayesian information criterion (BIC) were used to estimate and analyze the degree of fitting of the model. The APC analysis was done using Stata 15.0 software (College Station, TX, USA).

### Ethics approval and consent to participate

The study protocol was approved by the Ethical Review Board of Renmin Hospital (ID: WDRY2019–K034) *in accordance with the Declaration of Helsinki.*

### Informed consent

The need for informed consent, according to national legislation, was waived by the Ethical Review Board of Renmin Hospital because this was a retrospective cohort study.

## Supplementary Information

Supplementary Information.

## Data Availability

All data analyzed during this study are included in this article.
