# Supplementary material for: Secular trends and age-period-cohort effect on adverse perinatal outcomes in Hubei, China (2011–2019)
Source: Sci Rep. 2022 Dec 29;12:22558. doi: 10.1038/s41598-022-27194-8 (PMC9800403; doi:10.1038/s41598-022-27194-8)
Supplement: Supplementary file 1 — Supplementary Information. [file 41598_2022_27194_MOESM1_ESM.docx]

| **Variables and segments** | **Year** | **APC (95% CI)** |
| --- | --- | --- |
| **HDP** |  |  |
| Trend1 | 2011-2013 | 4.0 (-13.2, 24.6) |
| Trend2 | 2013-2016 | -5.5 (-21.1, 13.2) |
| Trend3 | 2016-2019 | 10.2 (0.7, 20.6)* |
| AAPC (95% CI) | 2011-2019 | 2.5 (1.1, 3.9)* |
| **Abnormal placentation** |  |  |
| Trend1 | 2011-2014 | -7.1 (-23.0, 12.2) |
| Trend2 | 2014-2017 | 22.4 (-16.0, 78.4) |
| Trend3 | 2017-2019 | -13.6 (-40.7, 25.9) |
| AAPC (95% CI) | 2011-2019 | 1.2 (1.1, 2.1)* |
| **GDM** |  |  |
| Trend1 | 2011-2014 | 36.8 (-90.7, 119.7) |
| Trend2 | 2014-2017 | 118.2 (-99.0, 467.1) |
| Trend3 | 2017-2019 | -12.0 (-99.6, 191.3) |
| AAPC (95% CI) | 2011-2019 | 46.0 (-2.7, 119.0) |

**Table S1.** Trends of pregnancy complications (HDP, abnormal placentation, and GDM) in pregnant women using joinpoint regression analysis from 2011-2019. APC (annual percentage change), APPC (average annual percent change), CI (confidence interval), HDP (Hypertensive disorders of pregnancy), GDM (Gestational diabetes mellitus), *significantly different from 0 at alpha = 0.05 (p < 0.05)
